# Mapping *Aedes aegypti* indoor resting behavior reveals a preference vulnerable to householder-led vector control

**DOI:** 10.1093/pnasnexus/pgad226

**Published:** 2023-07-25

**Authors:** Luca Facchinelli, Bashir Alsharif, Jeff D Jones, Agnes Matope, Rôsangela M R Barbosa, Constância F J Ayres, Philip J McCall

**Affiliations:** Vector Biology Department, Liverpool School of Tropical Medicine, L15QA, Liverpool, UK; Departamento de Entomologia, Instituto Aggeu Magalhães, Fiocruz Pernambuco, 50.740-465 Recife (PE), Brazil; Vector Biology Department, Liverpool School of Tropical Medicine, L15QA, Liverpool, UK; Vector Biology Department, Liverpool School of Tropical Medicine, L15QA, Liverpool, UK; Departamento de Entomologia, Instituto Aggeu Magalhães, Fiocruz Pernambuco, 50.740-465 Recife (PE), Brazil; Departamento de Entomologia, Instituto Aggeu Magalhães, Fiocruz Pernambuco, 50.740-465 Recife (PE), Brazil; Vector Biology Department, Liverpool School of Tropical Medicine, L15QA, Liverpool, UK

**Keywords:** *Aedes aegypti*, resting behavior, insecticide, IRS, dengue

## Abstract

Many mosquito vectors rest inside human habitations, a behavioral trait that is exploited for vector control by indoor residual spraying (IRS) of interior walls with insecticide. Although IRS and its refined version targeted IRS are very effective against *Aedes aegypti*, they are expensive and logistically challenging to deliver in densely populated urban areas where outbreaks of dengue and other arboviruses are the greatest challenge. In experiments in Recife, Brazil, we set out to quantify the indoor resting behavior of *Ae. aegypti* at a level beyond that previously reported. We found that significantly more *Ae. aegypti* males, unfed and fed females visited the base of walls (height 0–20 cm, corresponding to 12.3% of the total wall surface) more frequently than upper wall areas, with the difference more pronounced at higher temperatures. When the lowest 20 cm of the walls was treated with an appropriate insecticide and colored black, we recorded up to 85% cumulative mortality after 24-h exposure in the experimental room. The findings are significant because feasibly, householders could treat this small and accessible target zone manually, without the need for visits by costly IRS teams or equipment, reducing insecticide use and enabling communities to actively protect their own indoor environment.

**Significance Statement**

*Aedes aegypti* mosquitoes transmit arboviruses that threaten public health worldwide. Control by indoor residual spraying (IRS), especially targeted IRS, is effective but costs limit widespread use. Building on previous studies, our experiments revealed that mosquitoes frequently visited the zone on walls 0–20 cm from the floor, 12% of the total wall area. At higher temperatures, the frequency of visits to this target zone increased. When the zone was insecticide-treated, up to 85% of released mosquitoes were killed in 24 h. The findings are significant because householders could treat this small discrete target zone manually, without IRS teams or equipment, reducing insecticide and enabling communities to actively protect their own indoor environment. Further evaluation is merited.

## Introduction

*Aedes aegypti* is widely distributed throughout the tropics where it is a major vector of human arboviruses including dengue, chikungunya, and Zika. Across Asia and in the Americas, females of this highly synanthropic diurnally active mosquito rest inside houses before and after blood feeding and oviposit in the many containers where its aquatic stages develop, in domestic and peridomestic settings (1). Adapting to human environments has enabled this species to thrive in urban settings (2), particularly in high-density residential areas of low- to middle-income countries (LMIC), where it is a serious vector of human diseases.

The peridomestic habit of *Ae. aegypti* has confounded efforts to develop safe, effective, and sustainable methods of vector control (3). Achieving the levels of coverage at the large scale necessary to impact on outbreaks requires treatment of every at-risk house within a relatively short time window, a task financially and logistically beyond the means of most urban authorities in endemic areas (4). There are no specific drugs or vaccines available for all but one of the arboviruses transmitted by *Ae. aegypti*, yellow fever, and vector control remains the primary means of mitigating the burden of disease. As a result, there are few other effective alternatives for controlling the spread of these arboviruses. The success of *Wolbachia*-based interventions offers unprecedented levels of control (5, 6) but for the foreseeable future, this approach is unlikely to be available for more than a limited fraction of those at risk of *Aedes*-borne diseases worldwide.

One method of effectively targeting *Ae. aegypti* is indoor residual spraying (IRS), which, in Asia and Latin America, exploits the preference of most populations of this mosquito outside to rest indoors by coating wall and ceiling surfaces with an aqueous residue of persistent insecticide that kills mosquitoes when they land (7–9). Potentially very effective, IRS requires skilled personnel using specialist spray equipment to enter homes and spray large quantities of insecticide on every indoor wall and ceiling. High coverage is required to impact, making IRS an expensive solution. Treating the entire interior surface area is not only costly and relatively slow, but the often malodorous residue is an undesirable consequence as frequently it is perceived as harmful by householders leading to high rates of refusal (10), reducing coverage further.

Recent studies on vector behavior have successfully refined the IRS approach, demonstrating that targeted IRS (TIRS), i.e. spraying only the walls and dark recesses below 1.5 m (4, 11, 12), is as effective as spraying the entire house. This improvement arose from studies that identified the indoor resting preferences of *Ae. aegypti* females in Latin American and Asian populations (7, 11, 13, 14). Targeted IRS enables reductions in both the quantity of insecticide and treatment time per house without compromising the impact (12, 15). However, while clearly a significant improvement, targeted IRS is still dependent on a centralized cadre of skilled workers with specialist equipment, and achieving sufficient coverage remains a challenge for many communities. What is called for and what inhabitants of those areas badly need is a method, whether IRS or another method, that each home can deploy independently to protect themselves in the event of an outbreak (3).

Building on the knowledge from previous studies (4, 11, 16), we explored *Ae. aegypti* indoor resting behavior in a series of simple experiments. Typically, our goal in such studies with other vectors has been to identify a “kill zone,” a small discrete and accessible location frequented by the vector, where insecticide residues can be safely and easily deployed to target the vector (17–19).

## Results

The tests and the purpose of each are summarized in Table S1. Mosquitoes used in all tests were from a field-derived colony originating in Recife, Brazil. Unfed females showed partial resistance to lambda-cyhalothrin in World Health Organization (WHO) cone tests (94.2% 24-h mortality, 113/120 unfed females) and no mortality in controls (data from experiment 3).

In a preexperiment, we released simultaneously, in 16 test replicates, 75 *Ae. aegypti* (25 each of adult males, unfed and fed females, 3–5 days post emergence) into the experimental room, the interior of which was entirely coated with nonsetting adhesive. We observed repeatedly that, after 1 h, the lowest wall surfaces 0–20 cm above the floor were capturing a higher number of mosquitoes than any of the other areas. This was true for all groups tested, but the differences were not statistically significant (Table S2). Trapping mosquitoes with adhesive measures only the mosquito's first point of contact (17) and though we suspected it was occurring, these results did not provide incontrovertible evidence that *Ae. aegypti* frequently moved between locations during resting periods. However, the trend observed conformed with earlier studies that had also reported greater numbers of *Ae. aegypti* females resting on lower wall surfaces (7, 11, 20, 21), and we explored it further.

### Experiment 1: visiting location preference

**Fig. 1.**
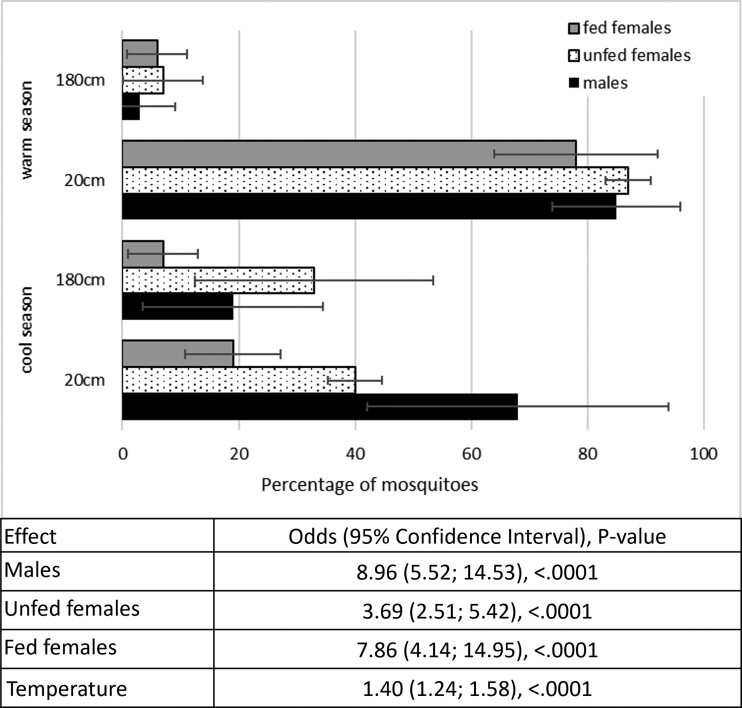
Graph shows the percentage and SD of mosquitoes of the different groups collected on the 0–20- vs. the 160–180-cm sticky strips in the cool (below) vs. the warm (above) seasons; table below reports the statistical results with temperature as a continuous variable, showing the OR, CI, and *P*-value for collecting mosquitoes on the 0–20- vs. the 160–180-cm adhesive strip.

We sought to confirm the preference for the lowest parts of walls by releasing *Ae. aegypti* for 24 h into the room setup as shown in Fig. S1A and B. From eight test replicates, four in the hot and four in the cool season, we recorded a mean percentage of recaptured mosquitoes ± SD of 96.0 ± 3.7 (males), 95.0 ± 4.1 (unfed females), and 95.5 ± 4.8 (fed females), after 24 h, and a mean percentage of mosquitoes recaptured on all sticky surfaces corresponding to 87.5 ± 9.9 (males), 83.5 ± 16.1 (unfed females), and 55.0 ± 32.6 (fed females). The graph in Fig. 1 shows how the number of mosquitoes collected on the lower strip was distributed between the two seasons. Statistical analysis considering temperature on a continuous scale presented in the same figure shows that there was a significantly higher probability that mosquitoes would be collected on the walls close to the floor than close to the ceiling (male odds ratio [OR] = 8.96; 95% CI: 5.52, 14.53; *P* < 0.01; unfed female OR = 3.69; 95% CI: 2.51, 5.42; *P* < 0.01; fed female OR = 7.86; 95% CI: 4.14, 14.95; *P* < 0.01). Notably, the likelihood of landing or being caught on the 0–20-cm strip compared with the 160–180-cm strip across all *Ae. aegypti* groups was 40% higher for every unit increase in room temperatures (1°C) (table in Fig. 1, OR = 1.40; 95% CI: 1.24, 1.58; *P* < 0.01).

### Experiment 2: residual insecticide test—lambda-cyhalothrin Cs

To investigate if this resting preference could be exploited to target insecticide delivery, we removed the higher sticky surface and replaced the low-level sticky surface with a white paper strip impregnated with the recommended dosage of insecticide residue (0.5 ml/m^2^ of microencapsulated lambda-cyhalothrin 2.5% [*w*/*w*], Demand 2.5 CS from Syngenta). Prior to testing, we ran the following system controls (see Materials and methods): control 1: 98.6% of released mosquitoes were collected alive 24 h post exposure, with no further mortality recorded after that point; control 2: 24-h mean mortality and SD of unfed females was 75% ± 14.1, and no mortality was detected in controls; and control 3: no mortality was recorded after 24-h exposure.

**Fig. 2.**
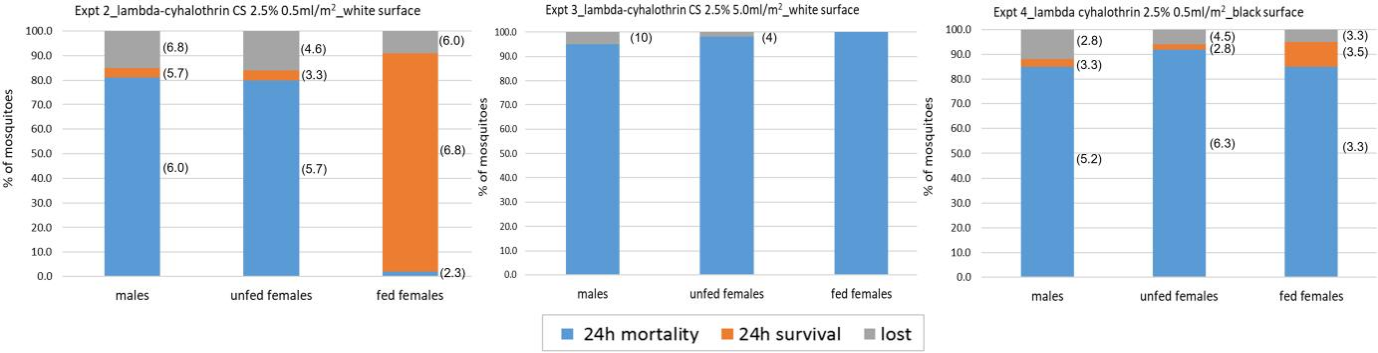
Mortality at 24-h post exposure for males, unfed and fed females in experiments 2–4. Left: lambda-cyhalothrin CS 2.5%, 0.5 ml/m^2^ on white surface. Center: lambda-cyhalothrin CS 2.5%, 5.0 ml/m^2^ on white surface. Right: lambda-cyhalothrin CS 2.5%, 0.5 ml/m^2^ on black surface. SD are shown in brackets alongside each outcome measure, added later to the graphs.

The mean recapture percentages and SD in the experiment were 85.0 ± 6.8 (males), 84.0 ± 4.6 (unfed females), and 91.0 ± 6.0 (fed females), after 24 h. Twenty-four- and 48-h mortalities (Fig. 2, left) were high for both males and unfed females (males: 81.0% ± 6.0 and 82.0% ± 5.2; unfed females: 80.0% ± 5.7 and 83.0% ± 3.8), but surprisingly low for fed females (2.0% ± 2.3 and 4.0% ± 5.7).

### Experiment 3: residual insecticide test—lambda-cyhalothrin CS 10×

To improve on the weak impact on fed females observed in experiment 2, we repeated the experiment using 10× (5.0 ml/m^2^) the recommended dosage of lambda-cyhalothrin CS. We also tested unfed vs. fed females in standard WHO cone tests to verify if they were equally impacted by insecticide exposure. Control 1: 92.0% (*n* = 69) of mosquitoes were collected alive with 1.4% mortality 24 h after exposure. Control 2: 24-h mortality was 100%, with 0% mortality in controls. Control 3: no mortality was recorded after 24-h exposure.

In the experimental room, we recorded a mean recapture percentage and SD of 95.0 ± 10.0 (males), 98.0 ± 4.0 (unfed females), and 100.0% (fed females) after 24 h. The mortality at 24 h post exposure was 100% for all groups (Fig. 2, center).

Results from WHO cone tests showed that the difference between 24-h median mortality rate in unfed (100%) vs. fed (70%) females was statistically significant (Fig. S2, *U* = 0.28; 95% CI: 0.16, 0.44; *P* < 0.01). No mortality in control cone tests 24 h after exposure was detected.

### Experiment 4: does the color of the treated surface affect mosquito mortality?

We repeated experiment 2 using the same methodology as before but changing the surface color of the treated paper to black.

Control 1: the recapture rate was 96.0% (*n* = 72), with a 24-h post exposure mortality of 1.4%. Control 2: 24-h mean mortality and SD of unfed females was 90% ± 15.1 and no mortality in controls. Control 3: not performed here as in experiment 3 as no effect of potential volatiles from the 10× dosage was detected on mosquitoes.

**Fig. 3.**
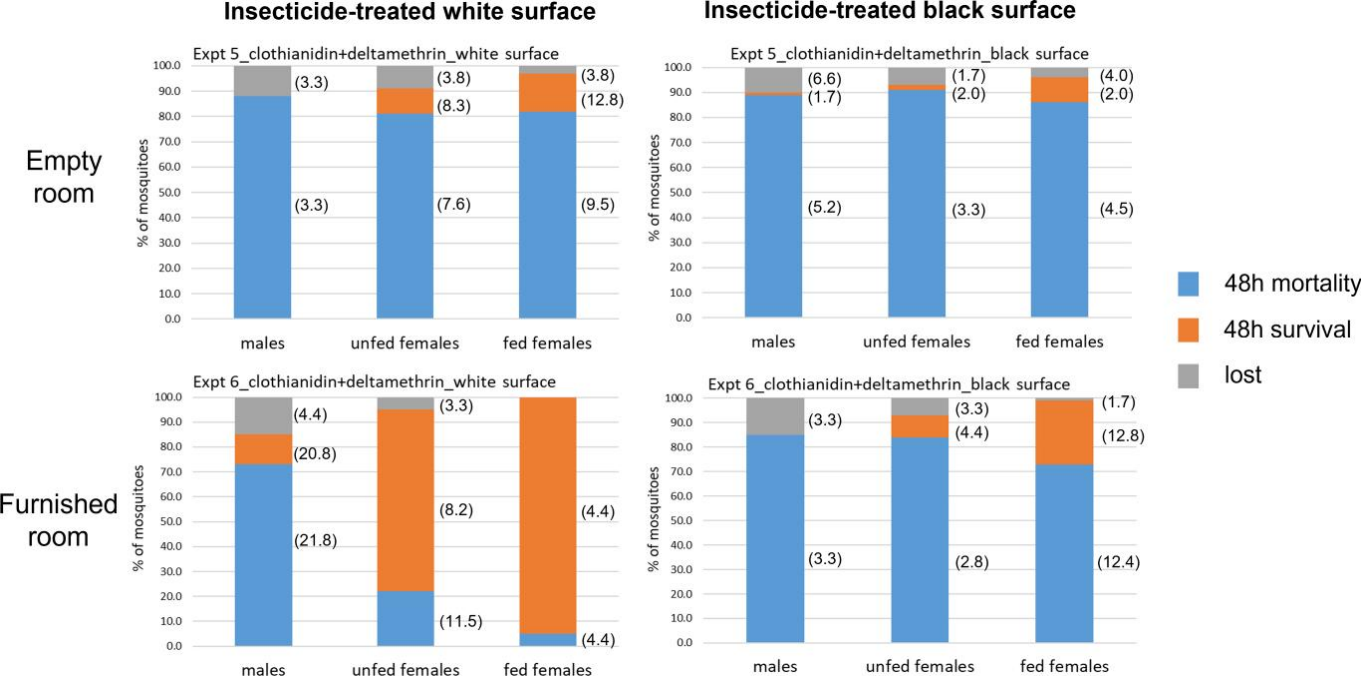
Mortality at 48-h post exposure for males, unfed females, and fed females in the unfurnished (top) vs. furnished (bottom) room using clothianidin (200 mg/m^2^) + deltamethrin (25 mg/m^2^) on white surface (top and bottom left) and black surface (top and bottom right). SD are shown in brackets alongside each outcome measure, added later to the graphs.

In the experimental room, we recorded a mean recapture percentage and SD of 88.0 ± 3.3 (males), 94.0 ± 5.2 (unfed females), and 95.0 ± 3.8 (fed females) after 24 h. Twenty-four- and 48-h mortalities (SD) were over 85% for all mosquitoes tested (males: 85.0% ± 5.2 and 88.0% ± 2.8; unfed females: 92.0% ± 6.3 and 93.0% ± 5.2; and fed females: 85.0% ± 3.3 and 85.0% ± 3.3) (Fig. 3, right). In fact, it would appear that changing the substrate color to black was almost as effective as raising by a factor of 10 the dosage of insecticide applied to the surface.

When comparing the 48-h mortality rates of experiment 2 vs. experiment 4, the fed females’ group results are significantly different, as the black strip treatment killed more mosquitoes than the white strip treatment (Table 1, OR = 136.00; 95% CI: 25.66, 720.93; *P* < 0.01).

**Table 1.** Statistical comparison among treatments and groups.

Experiment	Group	Color	Treatment	Room	Contrast	Model	Odds ratio	asymp.LCL	asymp.UCL	Null	*z* ratio	*P*-value	OR (95% CI), *P*-value
**2 and 4**	Fed		Lambda-cyhalothrin CS	Empty	Black/white	2	136.00	25.656	720.929	1	8.439	<0.001	136.00 (25.66, 720.93), <0.0010*
	Males		Lambda-cyhalothrin CS	Empty	Black/white	2	1.61	0.507	5.109	1	1.181	1.0000	1.61 (0.51, 5.11), 1.0000
	Unfed		Lambda-cyhalothrin CS	Empty	Black/white	2	2.72	0.700	10.576	1	2.113	0.4154	2.72 (0.70, 10.58), 0.4154
**5**	Fed		Clothianidin + deltamethrin	Empty	Black/white	1	1.35	0.443	4.103	1	0.770	1.0000	1.35 (0.44, 4.1), 1.0000
	Males		Clothianidin + deltamethrin	Empty	Black/white	1	1.10	0.309	3.934	1	0.222	1.0000	1.10 (0.31, 3.93), 1.0000
	Unfed		Clothianidin + deltamethrin	Empty	Black/white	1	2.37	0.687	8.190	1	1.997	0.5503	2.37 (0.69, 8.19), 0.5503
**2 and 4 vs. 5**	Fed	White		Empty	Lambda-cyhalothrin CS/clothianidin + deltamethrin	2	0.01	0.002	0.047	1	−8.195	<0.001	0.01 (0.00, 0.05), <0.0010*
	Males	White		Empty	Lambda-cyhalothrin CS/clothianidin + deltamethrin	2	0.62	0.196	1.971	1	−1.181	1.0000	0.62 (0.20, 1.97), 1.0000
	Unfed	White		Empty	Lambda-cyhalothrin CS/clothianidin + deltamethrin	2	1.15	0.398	3.293	1	0.368	1.0000	1.15 (0.40, 3.29), 1.0000
	Fed	Black		Empty	Lambda-cyhalothrin CS/clothianidin + deltamethrin	2	0.92	0.292	2.918	1	−0.201	1.0000	0.92 (0.29, 2.92), 1.0000
	Males	Black		Empty	Lambda-cyhalothrin CS/clothianidin + deltamethrin	2	0.91	0.254	3.231	1	−0.222	1.0000	0.91 (0.25, 3.23), 1.0000
	Unfed	Black		Empty	Lambda-cyhalothrin CS/clothianidin + deltamethrin	2	1.31	0.292	5.915	1	0.520	1.0000	1.31 (0.29, 5.92), 1.0000
**6**	Fed		Clothianidin + deltamethrin	Furnished	Black/white	1	51.37	11.877	222.183	1	7.707	<0.001	51.37 (11.88, 222.18), <0.0010*
	Males		Clothianidin + deltamethrin	Furnished	Black/white	1	2.10	0.748	5.869	1	2.059	0.4740	2.10 (0.75, 5.87), 0.4740
	Unfed		Clothianidin + deltamethrin	Furnished	Black/white	1	18.61	6.555	52.856	1	8.027	<0.001	18.61 (6.55, 52.86), <0.0010*
**5 vs. 6**	Fed	Black	Clothianidin + deltamethrin		Empty/furnished	1	2.27	0.797	6.480	1	2.244	0.2983	2.27 (0.80, 6.48), 0.2983
	Males	Black	Clothianidin + deltamethrin		Empty/furnished	1	1.43	0.423	4.825	1	0.838	1.0000	1.43 (0.42, 4.82), 1.0000
	Unfed	Black	Clothianidin + deltamethrin		Empty/furnished	1	1.93	0.541	6.859	1	1.479	1.0000	1.93 (0.54, 6.86), 1.0000
	Fed	White	Clothianidin + deltamethrin		Empty/furnished	1	86.56	19.095	392.355	1	8.457	<0.001	86.56 (19.09, 392.35), <0.0010*
	Males	White	Clothianidin + deltamethrin		Empty/furnished	1	2.71	0.909	8.089	1	2.616	0.1066	2.71 (0.91, 8.09), 0.1066
	Unfed	White	Clothianidin + deltamethrin		Empty/furnished	1	15.11	5.528	41.330	1	7.735	<0.001	15.11 (5.53, 41.33), <0.0010*

Results based on two separate logistic regression models.

OR, odds ratio

*Significant at 5% significance level.

### Experiment 5: targeting *Ae. aegypti* with clothianidin and deltamethrin

This experiment is a repeat of experiments 2 and 4, using a commercial insecticide containing 50% clothianidin and 6.25% deltamethrin (Fludora Fusion Bayer, Leverkusen), applied at a target dose rate of 200 mg/m^2^ (clothianidin) and 25 mg/m^2^ (deltamethrin).

Control 1: The percentage of recaptured mosquitoes was 92.0% (*n* = 69), with 2.9% mortality at 24 h after exposure. Control 2: 24-h mortality for both unfed and fed females in the WHO cone test was 100%, with 0% mortality in controls for both black and white papers. Control 3: 1.3% (*n* = 1) mortality was recorded after 24-h exposure.

In the experimental room, we recorded in the white paper treatment a mean recapture percentage and SD of 88.0 ± 3.3 (males), 91.0 ± 3.8 (unfed females), and 97.0 ± 3.8 (fed females) while in the black paper, treatment was 90.0 ± 7.7 (males), 93.0 ± 2.0 (unfed females), and 98.0 ± 4.0 (fed females) after 24 h. Twenty-four- and 48-h mortalities in the white paper treatment were 85.5% ± 5.0 and 88.0% ± 3.3 for males, 77.0% ± 7.6 and 81.0% ± 7.6 for unfed females, and 67.0% ± 13.6 and 82.0% ± 9.5 for fed females (48-h mortality shown in Fig. 3 top left). The 24- and 48-h mortalities in the black paper treatments were above 85% for all groups of *Ae. aegypti* tested: 89.0% ± 5.2 for males (both 24- and 48-h mortalities), 89.0% ± 4.4 and 91.0% ± 3.3 for unfed females, and 85.0% ± 5.2 and 86.0% ± 4.5 for fed females (48-h mortality is shown in Fig. 3 top right).

No statistically significant differences were observed in 48-h mortality among the three mosquito groups in the white vs. black paper treatments (Table 1, male OR = 1.10; 95% CI: 0.31, 3.93; *P* = 1.0000; unfed female OR = 2.37; 95% CI: 0.69, 8.19; *P* = 0.5503; fed female OR = 1.35; 95% CI: 0.44, 4.1; *P* = 1.0000). Interestingly, when we compare the results obtained using lambda cyhalothrin CS vs. clothianidin and deltamethrin (experiments 2 and 4 vs. 5), the pyrethroid only product proved to be less effective than the dual-ingredient product against fed females when using the white strips (Table 1, OR = 0.01; 95% CI: 0.00, 0.05; *P* < 0.01). No statistically significant differences among the two products were detected when employing the treated black paper strips (Table 1).

### Experiment 6: targeting *Ae. aegypti* in complex environments

Here we assessed if, in a slightly more heterogeneous room with multiple resting choices, the treated surface would still be attractive enough to lure and kill mosquitoes.

Control 1: the percentage of recaptured mosquitoes was 92.0% (*n* = 69) of which 5.8% died 24 h after exposure. Control 2: 100% of both unfed and fed females died in the WHO cone test with white and black papers, with 0% mortality recorded in controls. Control 3: 1.3% of mosquitoes spending 24 h in a cage inside the experimental room died after 24 h (*n* = 1).

In the experimental room, we recorded a mean recapture percentage and SD after 24 h in the white paper treatment of 85.0% ± 5.0 (males), 95.0 ± 3.8 (unfed females), and 100.0 (fed females); in the black paper treatment, they were 85.0% ± 3.8 (males), 95.0 ± 5.0 (unfed females), and 99.0% ± 2.0 (fed females) after 24 h. Twenty-four- and 48-h mortalities were identical for the white paper treatments at 73.0% ± 21.8 (males), 22.0% ± 11.5 (unfed females), and 5.0% ± 4.4 (fed females) (48-h mortality in Fig. 3 bottom-left). Twenty-four- and 48-h mortalities in the black paper treatment were also identical and exceeded 70% for all groups: 85.0% ± 3.3 (males), 84.0% ± 2.8 (unfed females), and 73.0% ± 12.4 (fed females) (48-h mortality in Fig. 3 bottom-right).

When comparing the 48-h mortality in the white vs. black paper strip treatments, a statistically significant difference for the unfed and the fed females’ groups was observed, with the treated black strip showing an increased likelihood to kill female mosquitoes compared with the white strip treatment (Table 1, unfed female OR = 18.61; 95% CI: 6.55, 52.86; *P* < 0.001; fed female OR = 51.37; 95% CI: 11.88, 222.18; *P* < 0.001).

Interestingly, comparing the results obtained using clothianidin and deltamethrin in the white paper treatments (experiment 5 vs. 6), the 48-h mortality results in the empty room were significantly higher than in the furnished room (Table 1, unfed female OR = 15.11; 95% CI: 5.53, 41.33; *P* < 0.01; fed female OR = 86.56; 95% CI: 19.09, 392.35; *P* < 0.01). No significant differences were found for experiments with the black strip treatments in the different rooms. Ultimately, the method will require validation in housing and rooms that reproduce fully the complexity within homes in target communities.

## Discussion

Our study demonstrated that in controlled conditions, resting *Ae. aegypti* adults consistently visited a significantly smaller area at the lowest level of the walls during a 24-h period than was previously known. Duration of contact with this wall surface occurred for long enough to pick up a lethal dose of insecticide, and though this region amounted to only 12.3% of the total wall area, spraying here alone could kill over 85% of *Ae. aegypti* in the room within 48 h, especially when the treated surface is black in color. The increase in content in the furnished room reduced the impact (presumably by reducing landing rates or duration of rest, when the target zone was white in color) but impact was restored by switching to a black impregnated surface, with 85% of males, 84% of unfed females, and 73% of blood-fed females killed within 48 h, a result comparable to that obtained in the empty experimental room.

This discovery has considerable value for *Ae. aegypti* control. If confirmed in the field, it demonstrates for the first time that sufficient residual insecticide to treat and protect the home could be delivered independently by members of a household using insecticidal paint or a handheld aerosol can and following a simple instruction or guideline. This deliverable satisfies the long expressed (3, 22) need for a vector control method that can be mobilized rapidly in high-density urban areas during an outbreak of dengue, chikungunya, or Zika.

Previous research indicated that female *Ae. aegypti* habitually rest on walls and dark objects (13, 14, 23, 24), typically below 1.5–2.0 m in height (11, 20). Though little is known about how environmental cues drive this behavior or how vision and temperature might affect it, Dunbar et al. (16) showed that treating only these resting site locations was sufficient to control infestations of *Ae. aegypti* to an extent comparable to complete standard IRS. This enabled dramatic reductions in the quantity of insecticide required per house compared with TIRS, without reducing the impact. However, to a nonexpert, recognizing all possible resting sites is at the very least a challenging prospect when applying insecticide in different rooms within the highly complex environment of houses. Simple directions on how, where, and when to treat standard surfaces are required if householders are expected to safely and effectively apply treatments without supervision. Subject to validation in further field studies, it appears highly likely that a similarly high level of impact can be achieved using the simple method proposed here.

The available literature identifies some indoor locations where *Ae. aegypti* is most frequently collected (7, 11, 20, 21), but it is not clear if adults remain in the same spot for lengthy periods during completion of physiological processes such as the gonotrophic cycle (static resting behavior) as has been reported (7), or if they change resting location frequently over time, driven by metabolic needs or by external or environmental stimuli (dynamic resting behavior). A dynamic resting habit, compatible with the results of our experiments, would be expected to increase the probability of contact if residual insecticides were applied to restricted but habitually visited fractions of the interior surface, because sooner or later adults would be expected to land on the treated areas.

Results in experiment 1 demonstrated how the increased temperature during the warmer season resulted in mosquitoes being “pushed down” toward the floor where temperatures were lower (Fig. 1). In fact, it is likely that the higher temperatures in the warm season would intensify this behavior, potentially increasing the efficacy of treating the lower part of the walls close to the floor. The importance of microclimatic conditions in mosquito resting site selection has been discussed elsewhere (25), with many species preferring cooler and more humid refugia, primarily to avoid desiccation. While extreme temperatures have a detrimental effect on both the length of the gonotrophic cycle and egg production (26), the model of Carrington et al. (2013) showed that a 26°C diurnal temperature range with temperature fluctuations at 7.6°C amplitude led to a steady exponential growth of *Ae. aegypti* population, with a short gonotrophic cycle and high egg production (26). Hypothetically, dynamic resting behavior would boost fed females’ reproductive fitness allowing them to change resting site and optimize the microclimatic conditions needed for rapid blood digestion and increased egg production. Notably, the transmission of some arboviruses also peaks at environmental temperatures between 23 and 29°C (27).

In our studies, the combination of clothianidin (neonicotinoid) and deltamethrin (pyrethroid) was more effective than lambda-cyhalothrin (pyrethroid) in targeting *Ae. aegypti*. In experiment 5, the fed females were killed when surfaces were treated with clothianidin and deltamethrin or a 10× dosage of lambda-cyhalothrin, but the recommended dosage of lambda-cyhalothrin was ineffective against this group. This is likely to be the result of the partial pyrethroid resistance still present in the *Ae. aegypti* colony used in the study. The 10× lambda-cyhalothrin treatment suggested that fed females still visited the lower part of the walls but did not rest there for long enough to become intoxicated at the recommended dosage.

*Aedes aegypti* populations in urban areas worldwide are, with few exceptions, effectively resistant to pyrethroids (28), hence the importance of demonstrating that the method being reported here is also effective using a product with an additional nonpyrethroid insecticide, in this case a neonicotinoid. The *Ae. aegypti* used in this study were colonized from field collections performed 3 years ago in Recife and still show residual resistance to lambda-cyhalothrin in standard WHO cone tests. Notably, blood-fed females were more tolerant of lambda-cyhalothrin than unfed females, most likely resulting from the increased production of metabolic enzymes following the blood meal, many of which would contribute to the rapid degradation of the insecticide, a phenomenon first reported recently in the African malaria vector *Anopheles gambiae* s.l. (29).

This report describes how careful exploration of the indoor resting behavior of *Ae. aegypti* revealed a route to a simple, rapid, yet potentially very effective method for the control of domestic infestations of *Ae. aegypti.* Clearly, the experiments should be repeated in a range of contexts to confirm the existence of the underlying behavior and the efficacy of the methods used to exploit it. However, we suggest that its considerable potential for the control of this most intractable of vectors is already apparent. Validation in a range of contexts would meet the numerous calls for simple, safe, and affordable vector control methods for use against *Ae. aegypti* (3, 22) and contribute to more general hopes of cutting the quantities of insecticide used per house with reductions in costs, human exposure, waste, and environmental contamination (10). Householder-delivered IRS might also impact on other vectors of diseases like malaria, leishmaniasis, and Chagas disease vectors, where they were found to exhibit resting habits akin to those reported here.

The success of any community-led intervention will depend on any number of factors ranging from the correct delivery by those performing the task to a perception of real benefit throughout the community.

Householder-led delivery of insecticide carries the risk that insufficient residue is applied to the target surfaces, resulting in sublethal treatments that can potentially promote selection of resistance in the target population as has occurred following fogging (30). Community-based vector control interventions that target households require deep understanding of the social and spatial dynamics of domestic life, the needs, priorities, and lifestyles of residents (31, 32). Resistance management plans may have to tolerate higher background levels of resistance, in order to protect more of the population for longer periods.

Targeted IRS has enabled IRS to become a more cost-effective approach to dengue control and today and is recommended for dengue prevention by WHO, PAHO, and, from this year, SEARO and WPRO too, covering a vast area wherein a majority of the human population globe live, many under threat of dengue, and many in urban areas at the highest densities the world has ever seen. Until TIRS, IRS was a very expensive approach to dengue control. If the approach proposed here delivered by householders or vertical vector control programs were to prove an effective alternative to standard TIRS, its benefits in terms of cost-effectiveness and sustainability would be unprecedented.

## Materials and methods

The study was conducted in a 290 × 250 × 200 cm (*L* × *W* × *H*) experimental room located at the Instituto Aggeu Magalhães, Cidade Universitária, Recife (PE), Brazil (Fig. S1). The room was built within a metal cage and protected from the rain with a transparent corrugated roof. The internal plasterboard walls and ceiling were painted magnolia white, marked with a pale blue 20-cm grid that allows recording the position where mosquitoes land and get stuck when using sticky surfaces. The floor and ceiling of the room were not perfectly parallel to each other, resulting in the width of the 180–200-cm strip on the walls ranging from 15 to 20 cm. In preliminary tests only, the walls and ceiling of the experimental room were entirely coated with the adhesive Zapicol, Zapi S.p.A., Italy (https://zapigarden.it/prodotto/zapicol), a product extensively used on sticky traps to catch *Stegomyia* mosquitoes (33–35). When furnished (experiment 6), the experimental room was intended to mimic a bedroom, the room where most of these mosquitoes are collected in real houses (7, 36). Temperature and relative humidity (RH) were recorded every 5 min in each test at different heights in the room (0 and 180 cm in experiment 1, or 0, 50, 100, 150, and 200 cm in experiments 2–6) and outside of the room (Table S3) using Tinytag Ultra 2 dataloggers (Gemini Data Loggers Ltd, Chichester, West Sussex, PO19 8UJ, UK).

Mosquitoes employed in the tests came from the RecLab colony established from ∼1,000 mosquitoes collected in Recife in 1996 (37). The colony was enriched before the experiments started with *Ae. aegypti* eggs collected in ovitraps from the Recife urban area in 2017. WHO cone tests performed during the study showed that the colony was partially resistant to lambda-cyhalothrin.

Before experiments, we ran three system controls as follows:

System control 1: to ensure background mortality in the experimental hut did not exceed 10%, as recommended by WHO, and to confirm that no volatile toxins or other contaminants that might increase mortality were present, we released simultaneously 2–5-day-old males, unfed and fed females, 25 of each group, in the experimental room and collected them 24 h later. Endpoints: % recapture and 24/48-h mortality rates.
System control 2: to confirm that test mosquitoes were susceptible to the insecticide tested by running WHO cone tests on the treated material being tested. Five mosquitoes per cone were exposed to untreated paper strips (two cones) and treated paper strips (eight cones), for a total of 50 mosquitoes. Endpoint: mortality rate.
System control 3: to ensure there were no volatile insecticides or other toxins within the test room that could increase the mortalities observed. To do this, we suspended within the room a cage holding 75 adult *Ae. aegypti* (25 each of 3–5-day-old males, unfed females and fed females). Endpoint: mortality rate.

In a preexperiment, we released simultaneously 2–5-day-old males, unfed and fed females, 25 of each group, in the experimental room entirely coated with glue and collected them 1 h after the release for a total of 16 replicates. Mosquitoes were collected stuck where they landed on the walls and the ceiling and their position on the 20-cm grid was recorded.

### Experiment 1: visiting location preference

On the experimental room's internal walls, we attached 20-cm-wide transparent plastic strips at heights of 0–20 and 160–180 cm (Fig. S1B) and coated the surface of each with adhesive. We used two strips at different heights from the floor based on the results of preliminary tests, with the goals of (i) quantifying the resting behavior on the lower part of the wall on a 24-h time frame, giving the possibility to mosquitoes to land on different parts of the internal surfaces before being trapped on the sticky areas, and (ii) being able to compare the effect of temperature on resting height in the cool vs. the warm season. Three- to 5-day-old males, unfed and fed *Ae. aegypti* females (25 of each group) were introduced in the room in a plastic container and acclimatized for 30 min before being released. Mosquitoes were released when the lid was removed by a mechanism operated from outside the room. Mosquitoes were collected after 24 h free flying inside the room by means of a backpack aspirator and an electric racket or stuck on the adhesive. Gender, physiologic status, and position on the sticky strips were recorded. A total of eight replicates were performed, four during the cooler (May–June) and four during the warmer (October–November) months of the year.

### Experiment 2: residual insecticide test—lambda-cyhalothrin CS

White paper strips were impregnated with 0.5 mL/m^2^ of 2.5% (*w*/*w*) microencapsulated lambda-cyhalothrin CS, Demand 2.5 CS, from Syngenta. Mosquitoes were released into the room using the same protocol employed for experiment 1. Adults were collected 24 h after the release, dead on the floor, resting on the walls, or free flying, and were kept in a cage in the insectary to assess 24- and 48-h mortalities. Four replicates for each treatment are performed. To ensure no transfer of insecticide contaminated the room between experiments, a 20-cm-wide plastic sheet was applied as a layer between the paper strips and the wall and renewed before starting a different treatment.

### Experiment 3: residual insecticide test—lambda-cyhalothrin CS 10×

Due to the low mortality of fed females obtained in experiment 2, we repeated the test protocol with 10× (5.0 mL/m^2^) the recommended target dose rate of lambda-cyhalothrin CS to have indirect evidence of fed females’ behavior, assuming that the high dosage would kill or knock them down if they visit the treated surface also for very short time. We also ran standard WHO cone tests in the insectary with the recommended dosage of lambda-cyhalothrin CS to compare unfed vs. fed females’ susceptibility with this insecticide. A total of 300 females were tested, 150 of which were unfed and 150 fed. Five mosquitoes were employed in each cone for a total of 60 cones, and 30 individuals per each group were employed as controls.

### Experiment 4: does the color of the treated surface affect mosquito mortality?

Materials and methods were the same as described in experiment 2 with the exception that, here, the paper strips were black in color.

### Experiment 5: targeting *Ae. aegypti* with clothianidin and deltamethrin

Methodology was the same as described for experiment 2. Here the internal walls of the experimental room were fitted with 20-cm-wide white (four replicates) or black (four replicates) paper strips located at 0–20 cm height from the floor and impregnated with the recommended dosage of clothianidin and deltamethrin.

### Experiment 6: targeting *Ae. aegypti* in a lightly complex environment

The experimental room was furnished as shown in Fig. S1C. The total potential landing area had also increased by 27.5%, from 28.85 m^2^ in the empty room to 36.77 m^2^ in the furnished room. All other materials and methods were as described in experiment 5. Internal walls were lined with white (four replicates) or black (four replicates) paper strips impregnated with Fludora Fusion as described in experiment 5. Methodology was the same as described here for experiment 5.

### Statistical analysis

Descriptive statistics were generated using the number of observations, mean, and SD for continuous variables and the number and percentage of observations for categorical variables. A logistic regression model was employed to analyze data from experiment 1, with coordinate/landing strip (two categories—20- and 160-cm strips) as a dependent variable and temperature and group (three categories—males, unfed females, and fed females) as independent variables. The logistic regression model was also employed to analyze the 48-h mortality rates from experiments 2 to 6. The independent variables were group, color (two categories—black and white), treatment (two categories—lambda-cyhalothrin CS 1× and clothianidin and deltamethrin), room complexity (two categories—empty and furnished), and their possible interactions. Two models were considered: clothianidin and deltamethrin only data—(i) to estimate the room complexity effect within color and color effect within room complexity and (ii) to estimate the treatment effect within color and color effect within treatment on 48-h mortality. The binomial distribution was considered for all the fitted models, and the OR and their corresponding 95% CIs were generated. All comparisons were conducted with a group (males, unfed females, and fed females) and were extracted using the R “emmeans” package and PROC PLM in SAS (38, 39). The Bonferroni multiplicity adjustment procedure was employed to control for the probability of making false-positive findings. Statistical significance was set at 5%. Statistical analysis was performed using SAS software version 9.4, R statistical software version 4.1.2, and R Studio (40, 41). Mann–Whitney test has been employed to analyze the 24-h mortality in WHO cone test comparison of unfed vs. fed females exposed to lambda-cyhalothrin CS in experiment 3.

## Supplementary Material

pgad226_Supplementary_Data

## Data Availability

All study data are included in the article and/or Supplementary material.
