# Supplementary material for: Mapping Aedes aegypti indoor resting behavior reveals a preference vulnerable to householder-led vector control
Source: PNAS Nexus. 2023 Jul 25;2(7):pgad226. doi: 10.1093/pnasnexus/pgad226 (PMC10368326; doi:10.1093/pnasnexus/pgad226)

**Table S1.** Summary of the goals and methodology for the experiments carried out in the study.


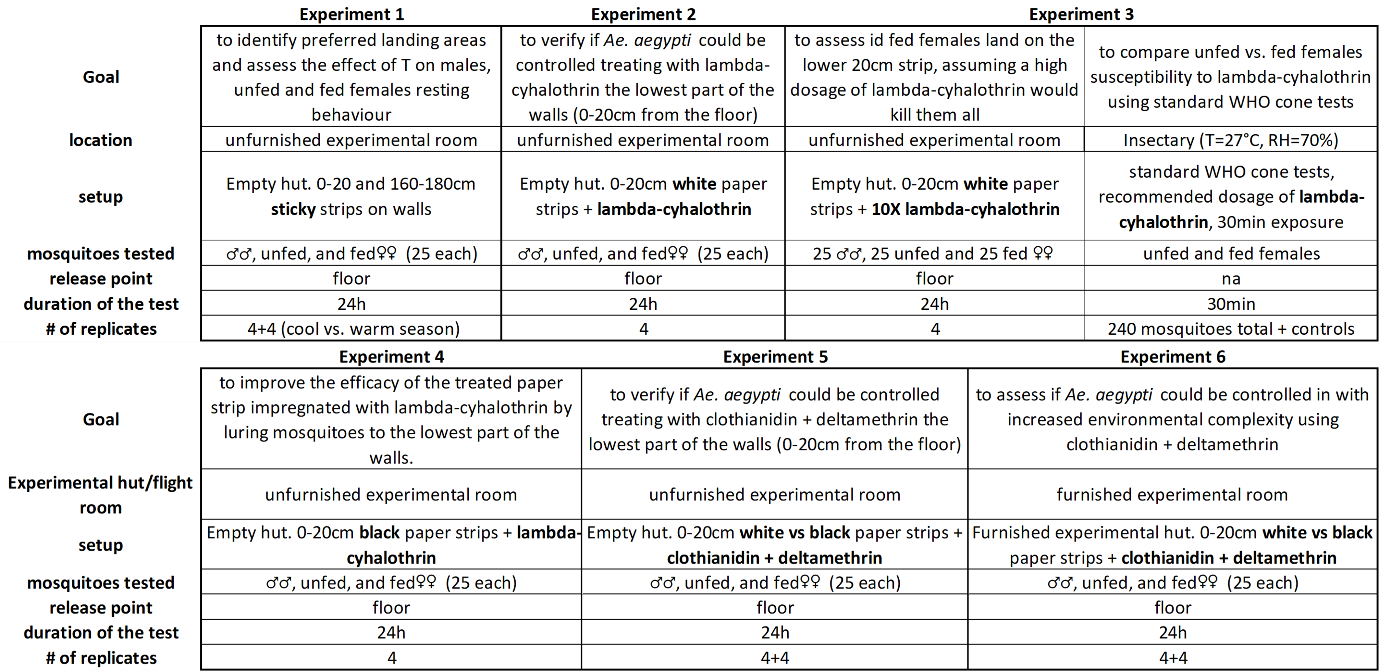


**Table S2.** Mean number of males, unfed and fed females collected at different heights from the floor (SD=standard deviation). Note: Shading values correspond to colour key range.


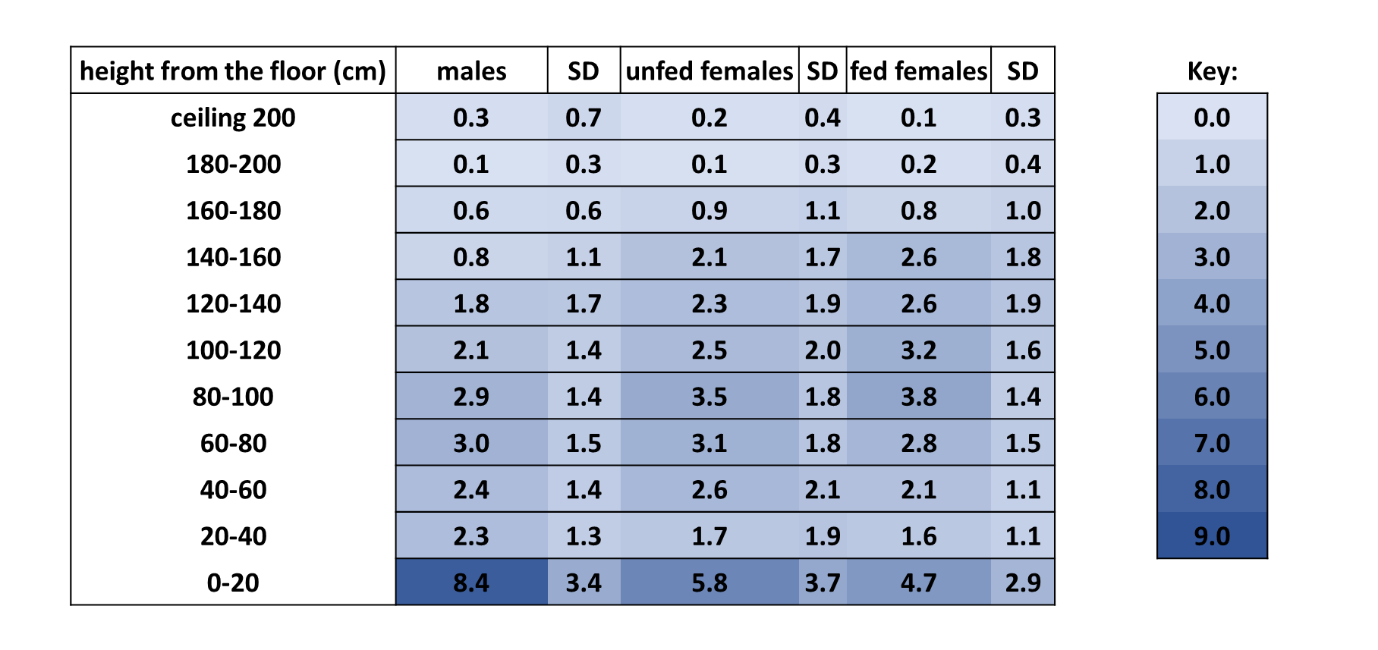


**Figure S1.** [A] external view; [B] exploded interior showing the internal walls (W1-4) and ceiling (Ce) overlaid with a 20cm grid; [C] furnished experimental room; . The positions of the two horizontal strips used in experiments are highlighted.


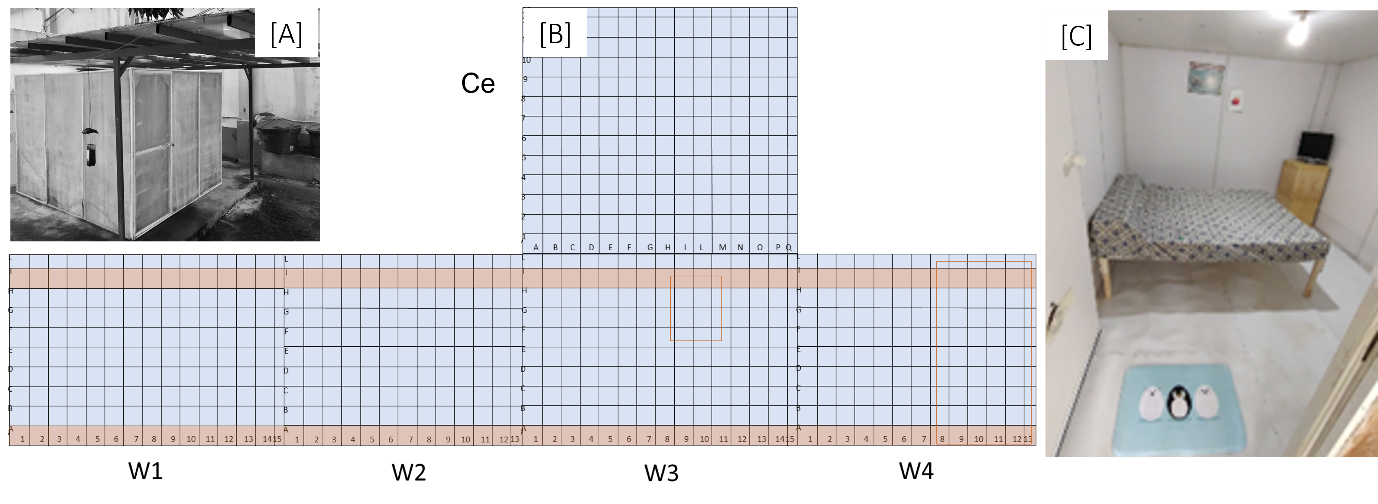


**Figure S2**. Box and Whisker plot for mortality rates of *Aedes aegypti* following 30 min exposure to lambda-cyhalothrin CS 2,5% 0.5ml/m^2^ in WHO cone tests.


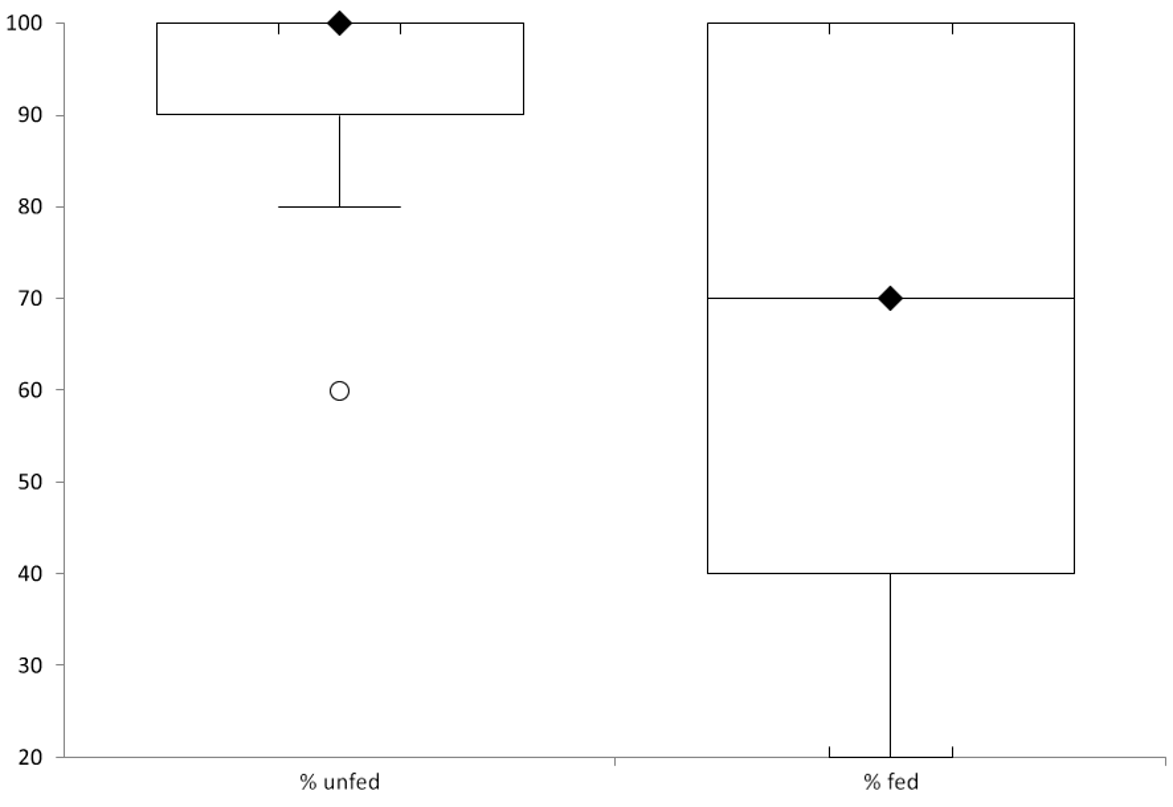


**Table S3.** Mean, Max and Min Temperature and RH and Standard Deviation (SD), recorded during each test at different heights in the room (0, 180 cm or 0, 50, 100, 150, 200 cm), and outside it (OUT).


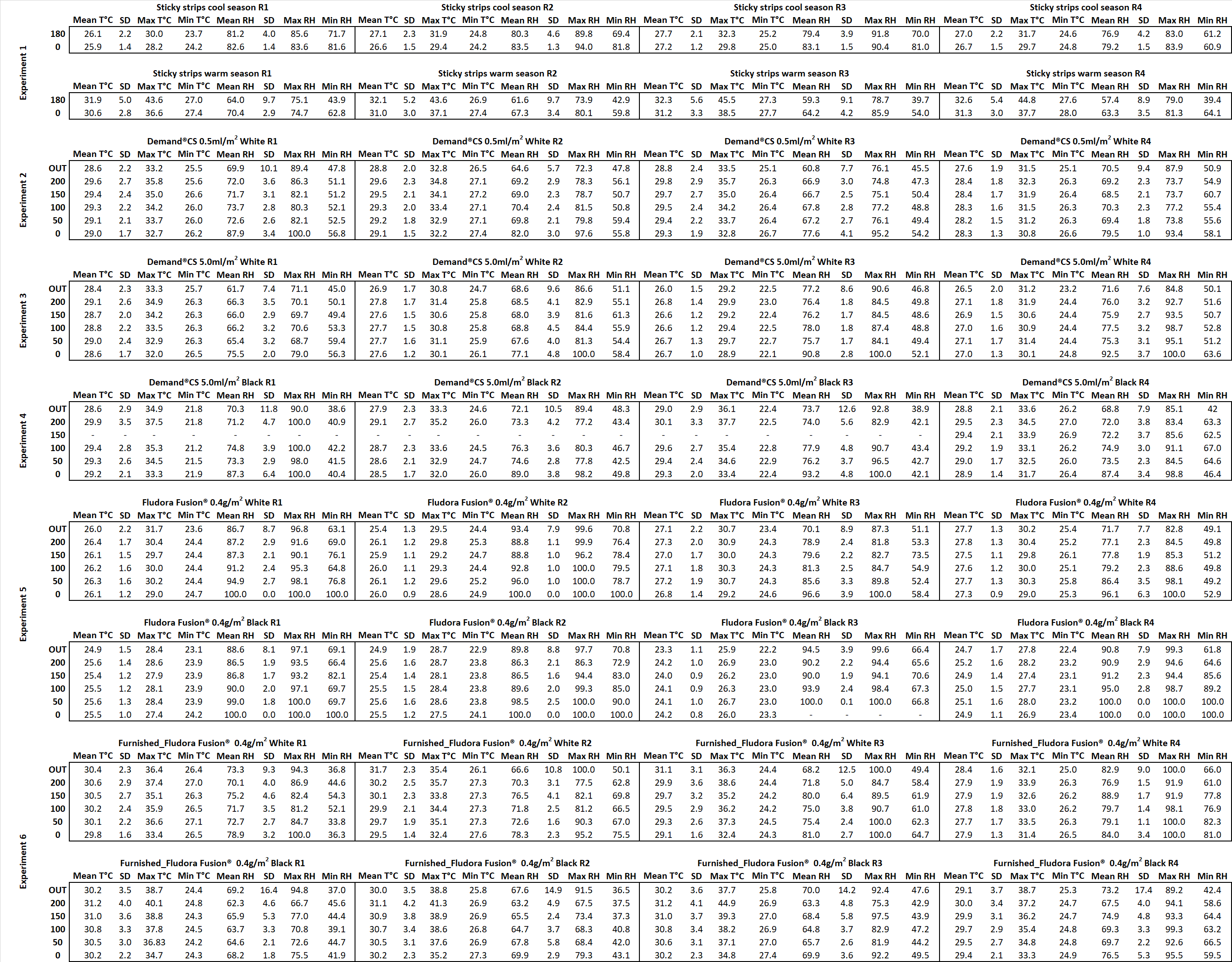

Supplement: pgad226_Supplementary_Data [file pgad226_supplementary_data.docx]
